# Reduced Kidney Function and Left Atrial Dilatation as Predictors of Incident Atrial Fibrillation in Patients With Hypertrophic Cardiomyopathy

**DOI:** 10.1002/joa3.70232

**Published:** 2025-11-25

**Authors:** Yuriko Tsumaya, Kenshi Hayashi, Toyonobu Tsuda, Akihiro Nomura, Yusuke Nakade, Mariko Oura, Takeo Yuno, Masako Nakata, Takako Terakami, Hiroyasu Oe, Megumi Oshima, Yoichiro Nakagawa, Takashi Kusayama, Shohei Yoshida, Hayato Tada, Mika Mori, Takeshi Kato, Kenji Sakata, Soichiro Usui, Noboru Fujino, Masayuki Takamura, Hajime Kanamori

**Affiliations:** ^1^ Department of Clinical Laboratory Kanazawa University Hospital Kanazawa Japan; ^2^ Department of Cardiovascular Medicine Kanazawa University Graduate School of Medical Sciences Kanazawa Japan; ^3^ School of Health Sciences, College of Medical, Pharmaceutical and Health Sciences, Kanazawa University Kanazawa Japan; ^4^ Department of Nephrology and Rheumatology Kanazawa University Kanazawa Japan; ^5^ Department of Emergency and Disaster Medicine Kanazawa University Graduate School of Medical Sciences Kanazawa Ishikawa Japan

**Keywords:** atrial fibrillation, chronic kidney disease, estimated glomerular filtration rate, hypertrophic cardiomyopathy, prediction

## Abstract

**Background:**

Atrial fibrillation (AF), the most frequently occurring sustained arrhythmia in patients with hypertrophic cardiomyopathy (HCM), is linked to poor quality of life and increased thromboembolic risk. Chronic kidney disease (CKD) and reduced kidney function are known cardiovascular risk factors; however, their contributions to new‐onset AF in patients with HCM remain unclear. Estimated glomerular filtration rate (eGFR) is a key marker for CKD management. This study aimed to elucidate the incidence of new‐onset AF and to identify predictive factors in patients with HCM.

**Methods:**

We analyzed 198 patients with HCM (121 men; mean age, 58 ± 17 years) without prior AF. The incidence and predictors of new‐onset AF were evaluated with a focus on kidney function and left atrial (LA) size. Cox proportional hazards modeling was used to assess the associations.

**Results:**

Impaired kidney function (eGFR < 60 mL/min/1.73 m^2^) was present in 35 patients (17.7%). Over a median follow‐up of 7.52 years, 43 patients (21.7%) developed new‐onset AF for an incidence rate of 2.8 per 100 person‐years. The multivariate analysis identified reduced eGFR and increased LA diameter (LAD) as independent predictors of AF. Kaplan–Meier curves showed a significantly higher cumulative AF incidence among patients with an eGFR ≤ 76.1 mL/min/1.73 m^2^ or an LAD ≥ 48.0 mm.

**Conclusions:**

Decreased kidney function and LA dilatation were significantly associated with new‐onset AF among patients with HCM. These findings suggest that this patient population requires closer monitoring for the early detection of AF.

## Graphical abstract

New‐onset atrial fibrillation (AF) occurred in 22% of patients with hypertrophic cardiomyopathy, corresponding to an incidence rate of 2.8 per 100 person‐years. Reduced kidney function and left atrial dilatation were independently associated with AF onset, indicating the need for closer monitoring and regular renal assessments in this population.

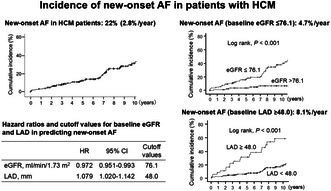

## 1 Introduction

Hypertrophic cardiomyopathy (HCM) is a genetic heart disorder characterized by progressive changes in morphology and pathophysiology due to lifelong left ventricular (LV) remodeling, with cardiovascular events occurring in relation to these pathophysiological alterations [1]. HCM has a family history that follows autosomal manifestations in 60% of cases; an estimated 40%–60% of these cases are caused by mutations in genes encoding sarcomeres and other cardiac component proteins [2, 3]. Atrial fibrillation (AF) is the most common arrhythmia in HCM [4]. A meta‐analysis of 33 clinical studies reported a 22.5% prevalence of AF in HCM and an incidence of new cases of AF of 3.1 per 100 patients/year [5]. Around 15% of HCM cases are complicated by AF presenting as heart failure (HF) and pulmonary edema [6].

Chronic kidney disease (CKD) is defined as abnormalities in kidney structure or function that persist for more than 3 months and affect one's health [7]. In routine clinical practice in Japan, kidney function is assessed using the Japanese Society of Nephrology estimated glomerular filtration rate (eGFR), which is based on serum creatinine levels, sex, and age. CKD increases the risk of cardiovascular disease in proportion to kidney impairment severity, resulting in poor outcomes. The phenomenon in which heart and kidney dysfunction are linked is known as cardiorenal syndrome. It has become clear that heart and kidney diseases are more likely to co‐occur and cross‐talk occurs between the two organs. A study examining mortality and cardiovascular disease incidence by kidney function found that both adverse events were higher in patients with an eGFR < 60 mL/min/1.73 m^2^ [8]. Several reports have stated that CKD and decreased kidney function are associated with an increased risk of incident AF [9, 10, 11]. However, the association between kidney dysfunction and incident AF in patients with HCM has not been fully elucidated.

This retrospective observational study aimed to determine the incidence and predictors of new‐onset AF among patients with HCM. Additional analyses were conducted to clarify the relationship between baseline eGFR or left atrial diameter (LAD) and the development of AF, the most commonly encountered arrhythmia in HCM.

## 2 Methods

### 2.1 Ethics Approval, Patient Consent, and Study Population

This study was conducted in accordance with the ethical standards of the Declaration of Helsinki and received approval from the Medical Ethics Committee of Kanazawa University Hospital. Between January 1991 and January 2015, a total of 292 patients diagnosed with HCM were enrolled and observed for at least 1 year at Kanazawa University Hospital. In adults, HCM was diagnosed based on echocardiographic or cardiac MRI findings of left ventricular hypertrophy, defined as a maximal wall thickness of 15 mm or more, in the absence of other identifiable causes. Left ventricular wall thickness of 13–14 mm was regarded as indicative of HCM when present in family members of an affected individual or when associated with a positive genetic test revealing a pathogenic or likely pathogenic variant, typically in a sarcomere‐related gene [12]. We excluded 54 patients with a history of AF and 40 for whom laboratory data were unavailable. A total of 198 patients were retrospectively evaluated.

### 2.2 Definitions of Risk Factors and Clinical Outcomes

Risk factors for AF [13] at baseline were defined as described previously [14]. Body mass index (BMI) was defined as a person's weight in kilograms divided by their height in meters squared and obesity was defined as BMI > 25 kg/m^2^. Hypertension was defined as a blood pressure ≥ 140/90 mmHg or the use of antihypertensive medications. Diabetes mellitus (DM) was defined by a fasting plasma glucose level above 126 mg/dL, a random plasma glucose level exceeding 200 mg/dL, a hemoglobin A1c greater than 6.5%, or the use of antidiabetic medication. HF was considered present if the patient had a prior hospitalization for HF, exhibited symptoms at baseline, or was receiving treatment for the condition. Anemia was defined as a hemoglobin level < 13.0 g/dL in men and 12.0 g/dL in women [15]. The eGFR was calculated using the Modification of Diet in Renal Disease Study equation modified for the Japanese population: 194 × Cr^‐1.094^ × age^‐0.287^ (× 0.739 for women) [16].

Electrocardiographic and echocardiographic measurements were obtained at the time of patient enrollment. Comprehensive evaluations with two‐dimensional and Doppler echocardiography were carried out using standard commercially available ultrasound devices. The anteroposterior diameter of the left atrium was obtained from the parasternal long‐axis view with two‐dimensional imaging. The peak left ventricular intracavitary pressure gradient was determined by continuous‐wave Doppler analysis. The primary endpoint of this analysis was new‐onset AF confirmed using electrocardiography or Holter electrocardiography.

### 2.3 Statistical Analysis

Continuous variables are expressed as mean ± standard deviation, and categorical variables are summarized as number and percentage (*n* %). Continuous variables were compared among the study groups using one‐way analysis of variance coupled with Dunnett's post hoc test, while categorical variables were compared using the chi‐squared test. Kaplan–Meier cumulative incidence curves were used to illustrate the occurrence of atrial fibrillation (AF), and differences between groups were assessed using the log‐rank test. Cox proportional hazards modeling was used to estimate adjusted hazard ratios (HRs) and corresponding 95% confidence intervals (CIs) for variables associated with AF events. Receiver‐operating characteristic (ROC) analysis was conducted to determine the most predictive cut‐off values for eGFR and LAD, and to evaluate their ability to discriminate incident AF during follow‐up. All statistical analyses were performed using SPSS Statistics 29.0.0 (SPSS Japan Inc., Tokyo, Japan) and JMP Pro version 10 (SAS Institute, Cary, NC, USA).

## 3 Results

### 3.1 Baseline Characteristics of the Study Population

The baseline characteristics of the 198 patients with HCM are shown in Table 1. The average patient age was 58 ± 17 years; 61.1% were men. Hypertension was observed in 50.0%, anemia in 15.7%, DM in 18.2%, and non‐sustained ventricular tachycardia (NSVT) in 26.3% of the subjects. HF was observed in 16.2%, and the average B‐type natriuretic peptide (BNP) level was 235.6 ± 334 pg/mL. The average eGFR was 78.2 ± 23.6 mL/min/1.73 m^2^: 35 of the 198 patients (17.7%) had impaired kidney function (eGFR < 60 mL/min/1.73 m^2^), 119 (60.1%) had mildly decreased kidney function (60 mL/min/1.73 m^2^ ≤ eGFR < 90 mL/min/1.73 m^2^), and 44 (22.2%) showed normal or high kidney function (eGFR ≥ 90 mL/min/1.73 m^2^). Electrocardiography revealed a prolonged QRS duration and high LV voltage. Echocardiography showed a maximal wall thickness of 17.8 ± 4.7 mm, LAD of 42.1 ± 6.9 mm, and LV ejection fraction (LVEF) of 68.1% ± 12%. An LV obstruction was observed in 21.2% of the study subjects.

**TABLE 1:** Baseline characteristics and comorbidities of the study patients.

	Total (*n* = 198)
Clinical characteristics	
Age, years	58 ± 17
Male, *n* (%)	121 (61.1)
BMI, kg/m^2^	23.8 ± 3.9
Systolic BP, mmHg	125 ± 20
Diastolic BP, mmHg	74 ± 12
Hypertension, *n* (%)	99 (50.0)
Diabetes, *n* (%)	36 (18.2)
Anemia, *n* (%)	31 (15.7)
Heart failure, *n* (%)	32 (16.2)
Coronary artery disease, *n* (%)	29 (14.6)
NSVT, *n* (%)	52 (26.3)
ICD/CRTD implantation, *n* (%)	24 (12.1)
Alcohol consumption, *n* (%)	89 (57.1)
Amiodarone use (%)	16 (8.1)
β blocker use (%)	100 (50.5)
ACEI/ARB use (%)	88 (44.4)
Clinical examination	
Hemoglobin, g/dL	14.0 ± 1.7
HbA1c, %	5.8 ± 1.2
eGFR, mL/min/1.73 m^2^	78.2 ± 23.6
BNP, pg/mL	235.6 ± 334
Electrocardiography	
HR, bpm	64 ± 10.6
PR, msec	170.8 ± 31
QRS, msec	103.1 ± 14
QTc, msec	430.8 ± 22
RV5 + SV1, mV	4.0 ± 1.6
Echocardiography	
MWT, mm	17.8 ± 4.7
LVDd, mm	46.2 ± 6.5
LAD, mm	42.1 ± 6.9
LVEF, %	68.1 ± 12
LVOT gradient > 30 mmHg, *n* (%)	42 (21.2)

Abbreviations: ACEI, angiotensin‐converting enzyme inhibitor; ARB, angiotensin II receptor blocker; BMI, body mass index; BNP, B‐type natriuretic peptide; CRTD, cardiac resynchronization therapy‐defibrillator; eGFR, estimated glomerular filtration rate; ICD, implantable cardioverter‐defibrillator; LAD, left atrial diameter; LVDd, left ventricular end‐diastolic diameter; LVEF, left ventricular ejection fraction; LVOT, left ventricular outflow tract; MWT, maximal wall thickness; NSVT, non‐sustained ventricular tachycardia.

### 3.2 Incidence of AF and Cox Proportional Regression Model for New‐Onset AF

During the observation period (median, 7.52 years), new‐onset AF was observed in 43 patients (21.7%), the incidence rate of 2.8 per 100 person‐years. The mean duration from the start of observation to the onset of AF was 5.33 ± 3.62 years. New‐onset AF was detected by single‐time 12‐lead electrocardiograms in 40 patients and by Holter electrocardiograms in 3 patients. Further analysis of the monitoring frequency during the observation period showed that the mean annual frequency of single‐time electrocardiograms was significantly higher in patients with new‐onset AF than in those without (5.5 ± 5.9 vs. 2.1 ± 2.1, *p* < 0.0001). Similarly, the annual frequency of Holter electrocardiograms was higher in patients with new‐onset AF (0.6 ± 0.6 vs. 0.2 ± 0.4, *p* < 0.0001). The Cox proportional hazards model was used to determine factors involved in the development of AF among patients with HCM. The unadjusted HR suggested that advancing age, HF, a reduced eGFR, NSVT, an increased LAD, an LV outflow tract pressure gradient > 30 mmHg, and implantable cardiac defibrillator/cardiac resynchronization therapy with defibrillator (ICD/CRTD) implantation were significantly associated with an increased risk of new‐onset AF (Table 2). In the multivariate analysis, a reduced eGFR (HR, 0.972; 95% CI, 0.951–0.993; *p* = 0.010) and increased LAD (HR, 1.079; 95% CI, 1.020–1.142; *p* = 0.008) were significantly associated with new‐onset AF.

**TABLE 2:** Cox proportional hazards model for the new‐onset of AF.

Variables	Univariate analysis		Multivariate analysis	
	HR (95% CI)	*p* value	HR (95% CI)	*p* value
Age, years	1.042 (1.017–1.069)	0.001	1.020 (0.990–1.050)	0.199
Male	0.743 (0.407–1.357)	0.334	0.868 (0.421–1.790)	0.702
Hypertension	1.276 (0.700–2.327)	0.427	0.571 (0.291–1.121)	0.103
Diabetes	1.561 (0.786–3.097)	0.203	1.080 (0.512–2.276)	0.839
Heart failure	3.722 (1.947–7.116)	< 0.001	1.083 (0.460–2.547)	0.855
Coronary artery disease	1.717 (0.823–3.580)	0.150	1.358 (0.621–2.970)	0.443
eGFR, mL/min/1.73 m^2^	0.958 (0.939–0.976)	< 0.001	0.972 (0.951–0.993)	0.010
PR > 200 ms	1.738 (0.833–3.626)	0.141	0.952 (0.421–2.155)	0.907
NSVT	2.391 (1.309–4.366)	0.005	1.829 (0.949–3.528)	0.071
LAD, mm	1.112 (1.061–1.166)	< 0.001	1.079 (1.020–1.142)	0.008
LVOT gradient > 30 mmHg	2.098 (1.120–3.927)	0.021	1.537 (0.780–3.030)	0.214
ICD/CRTD implantation	2.777 (1.424–5.414)	0.003		

Abbreviations: CRTD, cardiac resynchronization therapy‐defibrillator; eGFR, estimated glomerular filtration rate; ICD, implantable cardioverter‐defibrillator; LAD, left atrial diameter; LVOT, left ventricular outflow tract; NSVT, non‐sustained ventricular tachycardia.

### 3.3 Cut‐Off eGFR and LAD Values Predictive of New‐Onset AF

**FIGURE 1:**
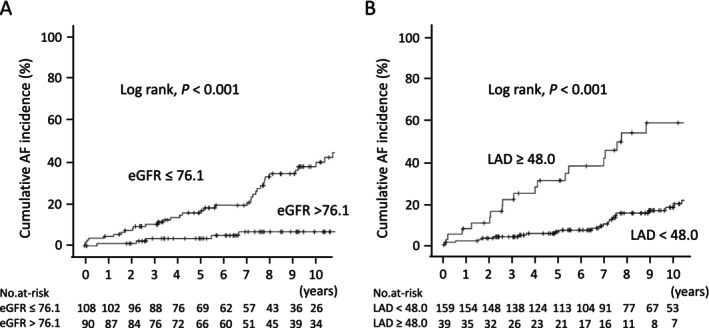
Cumulative incidence of new‐onset AF in patients with HCM. Kaplan–Meier curves for the incidence of AF during follow‐up period according to the level of baseline eGFR (A) or left atrial diameter (LAD) (B) dichotomized by the receiver‐operating characteristic curve derived cut‐off points.

For new‐onset AF, we constructed ROC curves and determined that the cut‐off value for eGFR was 76.1 mL/min/1.73 m^2^ (area under the curve, 0.71), while that for LAD was 48.0 mm (area under the curve, 0.69). We next examined whether these thresholds could predict new‐onset AF. The baseline characteristics of patients with HCM categorized according to these values are shown in Table 3. Regarding eGFR, the average age was significantly higher in patients with an eGFR ≤ 76.1 versus > 76.1 mL/min/1.73 m^2^. Diastolic BP, Hypertension, and HF were more frequently observed in patients with an eGFR ≤ 76.1 mL/min/1.73 m^2^. They also presented with higher BNP, longer PR duration, and larger LAD. New‐onset AF in patients with an eGFR ≤ 76.1 mL/min/1.73 m^2^ (*n* = 108) was observed in 33 cases for an incidence rate of 4.7/100 person‐years (Table 4). In contrast, the same was identified in only seven of 90 patients with an eGFR > 76.1 mL/min/1.73 m^2^, resulting in an incidence rate of 0.8 per 100 person‐years. As for LAD, average age and BMI were significantly higher in patients with an LAD ≥ 48.0 versus < 48.0 mm. HF, NSVT, and ICD/CRTD implantation were more frequently observed among patients with an LAD ≥ 48.0 mm. They also presented with lower eGFR, higher BNP, longer PR duration, larger LVDd, and lower LVEF. Among patients with LAD ≥ 48.0 mm (*n* = 39), 20 developed new‐onset AF, corresponding to an incidence rate of 8.1 per 100 person‐years (Table 4). In contrast, new‐onset AF was observed in 23 out of 159 patients with LAD < 48.0 mm, with an incidence rate of 1.8 per 100 person‐years. Kaplan–Meier analysis revealed that the cumulative incidence of AF was significantly higher in the groups with eGFR ≤ 76.1 mL/min/1.73 m^2^ (Log‐rank, *p* < 0.001) and LAD ≥ 48.0 mm (Log‐rank, *p* < 0.001) (Figure 1). The eGFR ≤ 76.1 mL/min/1.73 m^2^ at baseline could predict new‐onset AF in patients with HCM with 83.7% sensitivity, 54.2% specificity, 33.6% positive predictive value (PPV), and 92.3% negative predictive value (NPV). The LAD ≥ 48.0 mm at baseline could predict new‐onset AF in patients with HCM with 46.5% sensitivity, 87.7% specificity, 51.3% PPV, and 85.5% NPV.

**TABLE 3:** Baseline characteristics and comorbidities of patients with HCM categorized according to the level of eGFR or LAD.

	eGFR, mL/min/1.73 m^2^		*p* value	LAD, mm		*p* value
	≤ 76.1 (*n* = 108)	> 76.1 (*n* = 90)		< 48.0 (*n* = 159)	≥ 48.0 (*n* = 39)	
Clinical characteristics						
Age, years	64.3 ± 12.2	51.1 ± 18.2	< 0.001	57.2 ± 17.5	62.5 ± 11.2	0.004
Male, *n* (%)	66 (61.1)	55 (61.1)	1.000	98 (61.6)	23 (59.0)	0.760
BMI, kg/m^2^	24.1 ± 3.7	23.5 ± 4.2	0.334	23.4 ± 3.8	25.5 ± 3.8	0.003
Systolic BP, mmHg	127.1 ± 19.9	122 ± 19.9	0.067	124 ± 20.2	126 ± 19.4	0.615
Diastolic BP, mmHg	71.7 ± 11.3	76.7 ± 12.7	0.025	73.9 ± 12.4	72.1 ± 10.6	0.350
Hypertension, *n* (%)	66 (61.7)	33 (36.3)	< 0.001	80 (50.3)	19 (48.7)	0.858
Diabetes, *n* (%)	23 (21.3)	13 (14.4)	0.213	25 (15.7)	11 (28.2)	0.070
Anemia, *n* (%)	15 (13.9)	16 (17.8)	0.453	22 (13.8)	9 (23.1)	0.155
Heart failure, *n* (%)	27 (25.0)	5 (5.0)	< 0.001	19 (11.9)	13 (33.3)	0.001
NSVT, *n* (%)	34 (31.5)	18 (20.0)	0.068	35 (22.0)	17 (43.6)	0.006
ICD/CRTD implantation, *n* (%)	17 (15.7)	7 (7.8)	0.087	13 (8.2)	11 (28.2)	< 0.001
Clinical examination						
Hemoglobin, g/dL	14.0 ± 1.7	14.1 ± 1.8	0.633	14.1 ± 1.7	13.6 ± 1.9	0.096
HbA1c, %	5.8 ± 0.9	5.8 ± 1.4	0.890	5.8 ± 1.2	6.0 ± 0.8	0.137
eGFR, mL/min/1.73 m^2^	63.3 ± 9.5	96.0 ± 23.2	< 0.001	80.3 ± 24.5	69.6 ± 17.2	0.002
BNP, pg/mL	290.1 ± 390	170.1 ± 238	0.008	206.6 ± 316	353.6 ± 381	0.030
Electrocardiography						
HR, bpm	63.4 ± 9.9	65.4 ± 11.4	0.195	64.3 ± 11.0	64.3 ± 9.1	0.985
PR, msec	175.6 ± 32.0	164.9 ± 27.9	0.012	167.9 ± 29.5	182.3 ± 32.7	0.015
QRS, msec	103.6 ± 13.6	102.6 ± 13.9	0.613	102.3 ± 13.7	106.5 ± 13.3	0.090
QTc, msec	431.5 ± 22.7	430.5 ± 20.3	0.749	431.9 ± 21.8	427.3 ± 20.4	0.226
RV5 + SV1, mV	3.83 ± 1.6	4.19 ± 1.6	0.118	4.08 ± 1.5	3.65 ± 2.0	0.215
Echocardiography						
MWT, mm	18.0 ± 4.5	17.5 ± 5.0	0.536	17.8 ± 4.9	17.5 ± 3.9	0.644
LVDd, mm	46.6 ± 6.8	45.7 ± 6.0	0.332	45.3 ± 5.8	49.8 ± 7.7	0.001
LAD, mm	43.7 ± 6.7	40.2 ± 6.6	< 0.001	39.8 ± 5.3	51.7 ± 3.8	< 0.001
LVEF, %	67.9 ± 11.4	68.5 ± 12.1	0.720	69.7 ± 10.3	61.9 ± 14.6	0.003
LVOT gradient > 30 mmHg, *n* (%)	28 (25.9)	14 (15.6)	0.076	33 (20.8)	7 (21.2)	0.751

Abbreviations: BMI, body mass index; BNP, B‐type natriuretic peptide; CRTD, cardiac resynchronization therapy‐defibrillator; eGFR, estimated glomerular filtration rate; ICD, implantable cardioverter‐defibrillator; LAD, left atrial diameter; LVDd, left ventricular end‐diastolic diameter; LVEF, left ventricular ejection fraction; LVOT, left ventricular outflow tract; MWT, maximal wall thickness; NSVT, non‐sustained ventricular tachycardia.

**TABLE 4:** Incidence of AF according to eGFR or LAD level.

	eGFR, mL/min/1.73 m^2^		LAD, mm	
	≤ 76.1	> 76.1	< 48.0	≥ 48.0
No. of patients	108	90	159	39
No. of patients with AF (per 100 person‐years)	36 (4.7)	7 (0.8)	23 (1.8)	20 (8.1)

Abbreviations: eGFR, estimated glomerular filtration rate; LAD, left atrial diameter.

## 4 Discussion

This study aimed to clarify the relationship between kidney function and new‐onset AF development among patients with HCM. Our major findings are as follows. First, new‐onset AF was observed in 43 of 198 patients with HCM, an incidence of 2.8 per 100 person‐years. Second, a multivariate analysis using a Cox proportional hazards model demonstrated that a reduced eGFR and increased LAD were significantly associated with new‐onset AF among patients with HCM. AF was frequently observed during follow‐up in HCM patients with HCM and an eGFR ≤ 76.1 mL/min/1.73 m^2^ or LAD ≥ 48.0 mm at baseline with incidence rates of 4.7 and 8.1 per 100 person‐years, respectively.

### 4.1 Prevalence of CKD Among Patients With HCM

In this study, 17.7% of patients with HCM (mean age, 58 ± 17 years) also had comorbid moderate to severe kidney dysfunction (eGFR < 60 mL/min/1.73 m^2^). A previous study with 574 024 Japanese general adult participants (240 594 men; 333 430 women) showed an 11.4% prevalence of CKD with an eGFR < 60 mL/min/1.73 m^2^ among Japanese adults aged 50–70 years [17]. These data suggest that the incidence of moderate to severe kidney dysfunction is higher among patients with HCM than the general population. Several studies [18, 19] support our findings that a number of HCM cases featured comorbid moderate to severe kidney dysfunction. The baseline characteristics of patients with HCM in this study showed that HF was more frequently observed in patients with an eGFR ≤ 76.1 mL/min/1.73 m^2^. These patients are believed to have HF with preserved LVEF (HFpEF) since their average LVEF was 67.9% ± 11.4% (Table 3). Persistent cardiac dysfunction, including chronic congestive heart failure, may contribute to renal impairment, a condition classified as type 2 cardiorenal syndrome [20]. A previous study also reported that patients with chronic HFpEF appear to have survival rates similar to those with HF and reduced ejection fraction [21]. Observational clinical evidence suggests that preserved ejection fraction and elevated or normal blood pressure, leading to increased venous pressure, are key contributors to declining eGFR and activation of the renin‐angiotensin‐aldosterone system in the context of heart failure [22].

### 4.2 Frequency of New‐Onset AF in HCM

New‐onset AF was observed in 43 of 198 patients with HCM for an incidence rate of 2.8 per 100 person‐years. AF represents the most frequently encountered arrhythmia in individuals with HCM. HCM can lead to LV diastolic dysfunction and an increased LA load, making patients with HCM more prone to developing AF. According to a recent review, AF affects 20%–25% of individuals with HCM at some point in their lives, with an annual incidence rate of 2%–4% [23]. This result is consistent with the AF incidence rate of 2.8% in the present study.

In patients with HCM, the onset of AF has been linked to an unfavorable prognosis. The AF observed in HCM is associated with a higher risk of stroke and HF [24, 25]. We prospectively studied 1396 patients with nonvalvular AF using the Hokuriku‐Plus AF Registry and observed 79 cases of thromboembolism (incidence rate, 1.3 per 100 person‐years) and 192 cases of HF (incidence rate, 3.2 per 100 person‐years) during a median follow‐up of 5.0 years [25]. A Cox proportional hazards model demonstrated that concomitant HCM was significantly associated with the development of thromboembolisms and HF. Moreover, the rate of cardiovascular mortality in patients with HCM and AF is two‐ to three‐fold higher than that in those without AF [23, 26]. However, a recent study demonstrated better clinical outcomes in patients with HCM and AF, likely attributable to the use of modern therapeutic strategies such as catheter ablation and a broader range of antiarrhythmic medications [27].

In patients with HCM and AF, achieving sustained sinus rhythm remains challenging when relying solely on antiarrhythmic medications or catheter ablation. A recent review article showed that, in HCM, freedom from recurrent AF after catheter ablation alone was 50% at 1 year and 35% at 3 years [23]. It also showed that a combination of multiple ablations and continued antiarrhythmic drug use thereafter could be effective in up to 70% of patients with HCM in whom sinus rhythm was maintained for > 5 years [23]. Thus, in patients with HCM, it is desirable to identify the risk factors for AF onset and prevent its onset.

### 4.3 Impact of Reduced Kidney Function and LA Enlargement on New‐Onset AF in HCM

In this study, the multivariate analysis showed that eGFR and LAD were significantly associated with new‐onset AF. The eGFR ≤ 76.1 mL/min/1.73 m^2^ and the LAD ≥ 48.0 mm at baseline could predict new‐onset AF in patients with HCM with 83.7% and 46.5% sensitivity, 54.2% and 87.7% specificity, 33.6% and 51.3% PPV, and 92.3% and 85.5% NPV, respectively (Table 5). Regarding sensitivity and specificity, while AF prediction using eGFR may be characterized by fewer missed cases but a risk of overestimation, prediction based on LAD may conversely involve a likelihood of missed cases but reduced overprediction. The relatively high NPV of both parameters suggests that they may be useful for excluding the future onset of AF. Regarding PPV, of the 26 patients with both an eGFR ≤ 76.1 mL/min, 1.73 m^2^ and an LAD ≥ 48.0 mm, 17 developed AF. While the PPV of each parameter alone was limited, their combination resulted in an improved PPV of 65.4%.

**TABLE 5:** Diagnostic accuracy of incidence of AF according to eGFR or LAD level.

Parameter	eGFR, mL/min/1.73 m^2^	LAD, mm
Cut‐off	76.1	48.0
Sensitivity, %	83.7	46.5
Specificity, %	54.2	87.7
PPV, %	33.6	51.3
NPV, %	92.3	85.5

Abbreviations: eGFR, estimated glomerular filtration rate; LAD, left atrial diameter; NPV, negative predictive value; PPV, positive predictive value.

Previous studies demonstrated that the predictors of future AF in HCM include LA dilatation, increasing age, increased disease duration, HF symptoms, and the presence of LV late gadolinium enhancement [6, 12, 28, 29]. Impaired diastolic function in HCM causes LA enlargement and atrial tissue fibrosis, which can alter the conduction patterns and susceptibility to atrial reentry. LA dilatation is an established predictor of AF risk in HCM, and an LAD ≥ 45 mm, LA volume index ≥ 34 mL/m^2^, and LA end‐diastolic volume ≥ 118 mL are associated with the development of AF [6, 30, 31]. Consistent with previous reports, this study demonstrated a significant correlation between LA dilatation and new‐onset AF among patients with HCM.

Few studies have examined the relationship between impaired renal function and the development of new‐onset AF in patients with HCM. In our study, the incidence rate of AF was significantly higher in patients with an eGFR ≤ 76.1 mL/min/1.73 m^2^ (incidence rate, 4.7 per 100 person‐years) than in those with an eGFR > 76.1 (incidence rate, 0.8 per 100 person‐years). A baseline eGFR ≤ 76.1 mL/min/1.73 m^2^ was able to predict new‐onset AF with a sensitivity of 83.7% and specificity of 54.2%. CKD contributes to adverse cardiac remodeling, including LV hypertrophy and LV diastolic dysfunction, and is associated with increased cardiovascular risk, a condition referred to as type 4 cardiorenal syndrome. Notably, patients with CKD, particularly those undergoing hemodialysis, are at higher risk of developing AF. A previous Japanese population‐based cohort study demonstrated that the incidence of AF was higher among individuals with an eGFR < 60 mL/min/1.73 m^2^ (0.35% per year) than those with an eGFR of 60–89 mL/min/1.73 m^2^ (0.18% per year) or ≥ 90 mL/min/1.73 m^2^ (0.12% per year) [11]. Similarly, in the context of HCM, mild renal impairment appears to confer a higher risk of AF. Indeed, the ROC curve analysis of our cohort identified an eGFR cut‐off value of 76.1 mL/min/1.73 m^2^ for predicting new‐onset AF.

### 4.4 Study Limitations

There are several limitations associated with this study. First, this was a retrospective analysis conducted at a single center and involved a relatively limited number of patients. Further prospective research involving larger patient populations and extended follow‐up durations is needed. Second, the enrollment of patients with HCM depended entirely on the attending physicians. Of the 292 enrolled patients, 94 were excluded due to a history of AF or the unavailability of laboratory data. They may have caused selection bias. Third, we studied 198 patients without a history of AF; however, it is possible that asymptomatic patients with paroxysmal AF were included in the study group. Fourth, LAV or LAV index was available only in a limited number of patients; therefore, we used LAD in this study. Fifth, in the multivariate Cox proportional hazards analysis to identify factors associated with AF development in patients with HCM, the reliability of the results may be limited because of the relatively large number of explanatory variables compared with the number of events. The explanatory variables were selected based on established clinical risk factors that are known or have been previously reported to be associated with AF development, and were considered essential covariates for the analysis. In addition, variables that showed statistical significance in the univariate analysis were also included (Table 2). Finally, potential unmeasured bias, including the absence of detailed information on heart failure medications such as ACE inhibitors, ARBs, MRAs, and diuretics, may have influenced the findings of this study.

## 5 Conclusions

This retrospective study revealed that baseline eGFR and LAD have independent prognostic value in predicting future AF among patients with HCM. Patients with an eGFR ≤ 76.1 mL/min/1.73 m^2^ or a LAD ≥ 48.0 mm should be assessed more frequently for new‐onset AF using tools such as ambulatory electrocardiography and smart watch applications. Additionally, regular assessments of kidney function and effective kidney protection measures are desirable for preserving the quality of life and prognosis of patients with HCM.

## Funding

The authors have nothing to report.

## Ethics Statement

This study was approved by the Ethics Committee for Medical Research at Kanazawa University Hospital with an approval number of 111167.

## Consent

This study is an observational study based on medical records, and obtaining individual informed consent in advance was deemed difficult. Therefore, information about the study was posted in the clinic to ensure that potential participants had the opportunity to opt out.

## Conflicts of Interest

The authors declare no conflicts of interest.

## Data Availability

The participant data will not be shared.
